# Supplementary material for: Individual quality, insecure organizational attachment, and formalistic task completion: Social cognitive perspective
Source: PLoS One. 2024 Apr 1;19(4):e0301256. doi: 10.1371/journal.pone.0301256 (PMC10984427; doi:10.1371/journal.pone.0301256)
Supplement: S1 File — (DOCX) [file pone.0301256.s001.docx]

**The questionnaire of the study**

**Demographic information**

Gender: female; male

Age

Political status: CPC member; others

Education status: Undergraduate student; Master student; Ph.D student; Others

Major: Literature, history and art; Economics and management education; Science and Technology Military; Agronomy and Medicine; others

Cadre experience: yes; no

**Number Item**

IQ-K1 In the past six months, I often read various books.

IQ-K2 In the past six months, I have often engaged in reading or news activities in my free time.

IQ-K3 In the past six months, I often recharge and study in my free time.

IQ-K4 In the past six months, I often learn and expand other skills and experience in my free time.

IQ-P1 At work, I love hearing different ideas.

IQ-P2 At work, I like to think about problems.

IQ-P3 At work, I enjoy having philosophical discussions.

IQ-P4 At work, I feel comfortable being around a lot of people.

IQ-P5 At work, it is easy for me to make new friends.

IQ-P6 At work, I am good at dealing with social issues.

IQ-P7 I don't get easily bothered by things at work.

IQ-P8 At work, I relax most of the time.

IQ-P9 At work, I don't get angry easily.

IQ-P10 At work, I can empathize with other people's feelings.

IQ-P11 At work, I show concern when others have problems.

IQ-P12 At work, I have a keen eye for other people's emotions.

IQ-P13 At work, I will complete tasks in time.

IQ-P14 At work, when things are used up, I put them back in their proper place.

IQ-P15 At work, I like to be organized.

IA-As1 I worry that my organization doesn’t care for me.

IA-As2 I’m afraid of losing the “affection” and goodwill that my organization shows me.

IA-As3 I worry that my organization might want to replace me for someone else.

IA-As4 I often worry that my organization will not want me to remain as a member.

IA-At1 I prefer not to be too close to my organization.

IA-At2 I rarely turn to my organization in times of need.

IA-At3 I find it difficult to allow myself to depend on my organization.

**The context of formalistic tasks completion**

When you are notified that it is mandatory to attend and sign in for an academic conference that has nothing to do with your major, please evaluate and judge your actual performance based on your actual feelings and experiences as described below.

FTC1 I am able to perform the duties set out in the notice instructions.

FTC2 I can complete it at the level expected by my superiors.

FTC3 My performance will comply with the organization's performance regulations.

FTC4 I can perform all duties adequately.
