# Supplementary material for: Individual quality, insecure organizational attachment, and formalistic task completion: Social cognitive perspective
Source: PLoS One. 2024 Apr 1;19(4):e0301256. doi: 10.1371/journal.pone.0301256 (PMC10984427; doi:10.1371/journal.pone.0301256)
Supplement: Attachment — Submitted filename: Response to Reviewers.docx [file pone.0301256.s003.docx]

**Reviewers’ Comments and Our Revisions on**

“ Individual Quality, Insecure Organizational Attachment, and Formalistic Task Completion:

Social Cognitive Perspective”

We sincerely thank the editor for giving us chance to revise the paper and the anonymous reviewer’s insightful comments and suggestions, which have greatly helped us improve the quality of the paper. **We accepted all of the suggestions,** and have revised the manuscript according to the comments. The summary of comments and our revisions (highlighted with red color in the revised manuscript) are incorporated as follows.

**# Journal requirements**

1. When submitting your revision, we need you to address these additional requirements. Please ensure that your manuscript meets PLOS ONE's style requirements, including those for file naming.

**Our response:** Thank you. We have revised the paper in accordance with the requirements for submission to the PLOS ONE's style. The detail please see the file of “Revised Manuscript with Track Changes”.

2. Thank you for stating the following financial disclosure:

 [This work was funded by National Natural Science Foundation of China, grant number 71962021 and 72362028.]. 
Please state what role the funders took in the study.  If the funders had no role, please state: ""The funders had no role in study design, data collection and analysis, decision to publish, or preparation of the manuscript.""
If this statement is not correct you must amend it as needed. Please include this amended Role of Funder statement in your cover letter (4); we will change the online submission form on your behalf.

**Our response:** Thank you. The funders had no role in study design, data collection and analysis, decision to publish, or preparation of the manuscript. Meanwhile, we have amended Role of Funder statement in our cover letter.

3. Your ethics statement should only appear in the Methods section of your manuscript. If your ethics statement is written in any section besides the Methods, please move it to the Methods section and delete it from any other section. Please ensure that your ethics statement is included in your manuscript, as the ethics statement entered into the online submission form will not be published alongside your manuscript.

**Our response:** Thank for your reminder. We have checked where the ethical statements appear in our manuscript, and we ensure that our ethics statement is included in the manuscript and only appear in the Methods section.

4. Please include captions for your Supporting Information files at the end of your manuscript, and update any in-text citations to match accordingly. Please see our Supporting Information guidelines for more information: [http://journals.plos.org/plosone/s/supporting-information.](http://journals.plos.org/plosone/s/supporting-information.)

**Our response:** Thank you. We have no Supporting Information files in our manuscript.

5. Please review your reference list to ensure that it is complete and correct. If you have cited papers that have been retracted, please include the rationale for doing so in the manuscript text, or remove these references and replace them with relevant current references. Any changes to the reference list should be mentioned in the rebuttal letter that accompanies your revised manuscript. If you need to cite a retracted article, indicate the article’s retracted status in the References list and also include a citation and full reference for the retraction notice.
**Our response:** Thanks. We have reviewed our reference list to ensure that it is complete and correct. Specifically, we have no cited papers that have been retracted. And we have removed and update some references.

6. PLOS authors have the option to publish the peer review history of their article ([what does this mean?](https://journals.plos.org/plosone/s/editorial-and-peer-review-process#loc-peer-review-history)). If published, this will include your full peer review and any attached files.

**Our response:** NO

**Reviewer #1**

**Comment 1:** Overall it is good piece of research however it is recommended that authors to revise the document for the language and flow to improve readability.

**Our response and revision:** Thank for the suggestion and we accepted it. We have revised the document for the language and flow.

**Comment 2:** Use of terms like "I" and "We" should be avoided.

**Our response and revision:** Thank you. We accepted your suggestion and address the problem. The terms like “I” and “We” have removed in our manuscript.

**Comment 3:** Including few lines of conclusive discussions before given implications will be helpful in creating link between findings and implication.

**Our response and revision:** Thank you for your suggestion and we accepted it. We have added a paragraph to help explain the link between findings and implication (lines 412-422).

**Reviewer #2**

**Comment 1:** Overall manuscript need to be revised for it's correct use of language expressions.

**Our response and revision:**

**Our response and revision:** Thank for the suggestion and we accepted it. We have revised the manuscript for its correct use of language expressions.

**Comment 2:** The abstract needs to be revised in terms of comprehensively delivering the ideas.

**Our response and revision:** Thank you. We accepted your suggestion and improved the problem. Specifically, we have rewritten the part of abstract (lines 7-18).

**Comment 3:** Use of latest supporting reference is encouraged across the introduction and literature review sections.

**Our response and revision:** Thank you. We accepted your suggestion and improved the problem. We have updated the latest supporting reference across not only the introduction and literature review sections, but also the theories, hypotheses and discussion sections.

**Comment 4:** Authors need to present statistical support to justify the selection of the sample size.

**Our response and revision:** Thank you for your suggestion. We justify the selection of the sample size through the software of “GPower 3.1”. The sample size (602) of our study is larger than the sample size (210) calculated by GPower (Parameters are set as follows: effect size is 0.5; α err prob is 0.05; Power (1-β err prob) is 0.95).
