# Supplementary material for: Individual quality, insecure organizational attachment, and formalistic task completion: Social cognitive perspective
Source: PLoS One. 2024 Apr 1;19(4):e0301256. doi: 10.1371/journal.pone.0301256 (PMC10984427; doi:10.1371/journal.pone.0301256)
Supplement: Attachment — Submitted filename: Response to Reviewers.docx [file pone.0301256.s004.docx]

**Reviewers’ Comments and Our Revisions on**

“ Individual Quality, Insecure Organizational Attachment, and Formalistic Task Completion:

Social Cognitive Perspective”

We sincerely thank the editor for giving us chance to revise the paper and the anonymous reviewer’s insightful comments and suggestions, which have greatly helped us improve the quality of the paper. **We accepted all of the suggestions,** and have revised the manuscript according to the comments. The summary of comments and our revisions (highlighted with red color in the revised manuscript) are incorporated as follows.

**# Editor**

**Comment 1:** The author needs to polish the manuscript to increase the fluency of reading.

**Our response and revision:** We have improved the manuscript for its fluency of reading. The research team requested a native English speaking partner to assist us in improving the article.

**Comment 2:** The author needs to seek reputable journals to support AVE above 0.36 is acceptable and above 0.50 is recommended. Although this sentence is mentioned in the No.75 reference, there is a lack of attribution to its source.

**Our response and revision:** We accepted your suggestion and addressed the problem. Through our tracing of the literature, we added two studies from reputable journals to the "Reference", namely, **No. 76 reference** [Fornell, C., & Larcker, D. F. (1981). Evaluating structural equation models with unobservable variables and measurement error. *Journal of marketing research*, *18*(1), 39-50. page 47] and **No. 77 reference** [Lam, L. W. (2012). Impact of competitiveness on salespeople's commitment and performance. *Journal of Business Research*, *65*(9), 1328-1334.]. No. 75 reference was sourced from No. 76 reference.

In our study, the composite reliability of nine measures ranges from 0.666 to 0.850, which meets the acceptable level of 0.60 proposed by Fornell & Larcker (1981), and almost meets the level of 0.7 proposed by Hair et al. (2009). However, the AVE values for several variables are below the recommended threshold of 0.5. According to **No. 76 reference** (p. 46), “*Note that ρ_vc(η)_* (AVE) *is a more conservative measure than ρ_η_* (reliability) *alone, the research may conclude that the convergent validity of the construct is adequate, even though more than 50% of the variance is due to error*”. As the composite reliability of the nine constructs is well above the recommended level of 0.6, the internal reliability of the measurement items is regard as acceptable. Moreover, Long W. Lam (2012) we quoted as **No. 77 reference** published in the Journal of Business Research also quotes Fornell & Lacker (1981) as saying the above words too.

Hence, we made a revision in our manuscript as follows: “*The AVEs range between 0.383 and 0.544. Though some of them are below the usually recommended level of 0.5, the convergent validity of the corresponding constructs can be assumed adequate when the CRs are higher than 0.6 [73-77]*” (lines 365-368, p. 18).
