# Supplementary material for: Individual quality, insecure organizational attachment, and formalistic task completion: Social cognitive perspective
Source: PLoS One. 2024 Apr 1;19(4):e0301256. doi: 10.1371/journal.pone.0301256 (PMC10984427; doi:10.1371/journal.pone.0301256)
Supplement: Attachment — Submitted filename: Response to Reviewers.docx [file pone.0301256.s005.docx]

**Reviewers’ Comments and Our Revisions on**

“Individual Quality, Insecure Organizational Attachment, and Formalistic Task Completion:

Social Cognitive Perspective”

We sincerely thank the editor for giving us chance to revise the paper and the anonymous reviewer’s insightful comments and suggestions, which have greatly helped us improve the quality of the paper. **We accepted all of the suggestions,** and have revised the manuscript according to the comments. The summary of comments and our revisions (highlighted with red color in the revised manuscript) are incorporated as follows.

**Comment 1:**

“I compared the AVE values in the references you provided. There are two studies in its paper, and I can understand that AVE values are substandard for only one variable. I recommend that you use scientific methods to improve AVE, for example, item packaging. Here are the references I provided.”

**Our response and revision:**

Thank for the suggestion and we accepted it. We have revised the manuscript for improve the value of AVE.

We have carefully reviewed the literature you shared with us and studied the scientific method you proposed, namely, the item parceling (or item packaging). After learning about this method, we realized that although our study used multiple constructs in the questionnaire, each construct typically had only 3 to 5 items. In this case, we believe that the item parceling method may not be suitable for improving our AVE values. During this period, we also learned more scientific methods for data cleaning, and as a result, we re-cleaned the data, which improved the AVE values. The reliability and validity of the data have also been re-assessed and are reflected in Tables 1, Tables 2, and Tables 3 in the "Methodology" section of the paper. Additionally, after re-cleaning the data, we identified two new findings regarding the moderating effects, which are highlighted in red in the "Results" section of the paper.

Once again, we appreciate your advice, which has greatly enhanced the quality of our paper.
